# Happy and sad music acutely modulate different types of attention in older adults

**DOI:** 10.3389/fpsyg.2023.1029773

**Published:** 2023-01-26

**Authors:** Nicholas Dovorany, Schea Brannick, Nathan Johnson, Ileana Ratiu, Arianna N. LaCroix

**Affiliations:** ^1^College of Graduate Studies, Midwestern University, Glendale, AZ, United States; ^2^College of Health Sciences, Midwestern University, Glendale, AZ, United States; ^3^College of Health Solutions, Arizona State University, Tempe, AZ, United States; ^4^Department of Speech, Language, and Hearing Sciences, College of Health and Human Sciences, Purdue University, West Lafayette, IN, United States

**Keywords:** attention, aging, music, emotion, ant, alerting, executive control, orienting

## Abstract

Of the three subtypes of attention outlined by the attentional subsystems model, alerting (vigilance or arousal needed for task completion) and executive control (the ability to inhibit distracting information while completing a goal) are susceptible to age-related decline, while orienting remains relatively stable. Yet, few studies have investigated strategies that may acutely maintain or promote attention in typically aging older adults. Music listening may be one potential strategy for attentional maintenance as past research shows that listening to happy music characterized by a fast tempo and major mode increases cognitive task performance, likely by increasing cognitive arousal. The present study sought to investigate whether listening to happy music (fast tempo, major mode) impacts alerting, orienting, and executive control attention in 57 middle and older-aged adults (*M* = 61.09 years, SD = 7.16). Participants completed the Attention Network Test (ANT) before and after listening to music rated as happy or sad (slow tempo, minor mode), or no music (i.e., silence) for 10 min. Our results demonstrate that happy music increased alerting attention, particularly when relevant and irrelevant information conflicted within a trial. Contrary to what was predicted, sad music modulated executive control performance. Overall, our findings indicate that music written in the major mode with a fast tempo (happy) and minor mode with a slow tempo (sad) modulate different aspects of attention in the short-term.

## 1. Introduction

Attention is a multifaceted construct that moderates our interactions with the world (Petersen and Posner, 2012; Harvey, 2019). It also supports higher order cognitive processes such as working memory and executive functions (McCabe et al., 2010). There are several models that divide attention into distinct components (Sohlberg and Mateer, 1987; Mirsky et al., 1991), with one prominent model dividing attention into three subsystems: alerting, orienting, and executive control (Posner and Petersen, 1990). Alerting involves vigilance to perceive information, orienting is the ability to prioritize a specific aspect or location of a stimulus, and executive control is the ability to complete goal directed behavior while inhibiting distracting information (Posner and Petersen, 1990).

The Attention Network Test (ANT) simultaneously assesses alerting, orienting, and executive control using a cued-flanker task (Fan et al., 2002). In this computerized task, participants complete a flanker task (i.e., determine which direction a center arrow, in a series of five arrows, is pointing). A congruent trial occurs when all the arrows point in the same direction, an incongruent trial occurs when the center arrow points in the opposite direction of the flanking arrows, and a neutral trial occurs when the center arrow is presented with no flanking arrows. Participants are either cued to the onset of the flanker task with an alerting cue, the location of the flanker task with an orienting cue or provided with no cueing about when or where the upcoming flanker task will be presented. The efficiency of each attention subsystem is measured by comparing specific combinations of cues and flanker targets (described below in “attention network task”).

The ANT was initially developed and tested in younger adults (Fan et al., 2002), however it has since been used to test all three attentional subsystems in a variety of populations, including older adults (Gamboz et al., 2010; Zhou et al., 2011; Knight and Mather, 2013). Across several studies, older adults appear to be less alert, or benefit less from the alerting cue, than their younger counterparts (Jennings et al., 2007; Gamboz et al., 2010; Zhou et al., 2011; Knight and Mather, 2013; Kaufman et al., 2016; Williams et al., 2016). Similarly, for executive control, research indicates that executive control declines with increasing age. Compared to younger adults, older adults have greater difficulty resolving incongruent trials that contain conflict compared to neutral trials that are without conflict (Jennings et al., 2007; Gamboz et al., 2010; Mahoney et al., 2010; Zhou et al., 2011; Lu et al., 2016). In contrast to alerting and executive control, the ability to orient attention remains relatively stable with increasing age (Fernandez-Duque and Black, 2006; Jennings et al., 2007; Gamboz et al., 2010; Mahoney et al., 2010; Zhou et al., 2011). Alerting and executive control may be particularly susceptible to age-related decline, compared to orienting attention, because they are supported by the frontal lobes (among other regions; Roberts and Hall, 2008; Anderson et al., 2010; Petersen and Posner, 2012; Rinne et al., 2013). The frontal lobes atrophy as part of the normal aging process (Jernigan et al., 2001; Peters, 2006; Glisky, 2007; Zanto and Gazzaley, 2019). For instance, research shows that prefrontal cortical volume declines at a rate of approximately 5% per decade starting around the age of 40 (Svennerholm et al., 1997; Jernigan et al., 2001; Walhovd et al., 2005; Peters, 2006; Long et al., 2012). In contrast, more posterior regions, including regions associated with orienting attention in the parietal lobes (Corbetta and Shulman, 2002; Petersen and Posner, 2012), are less susceptible to cortical volume loss as a function of age (Trollor and Valenzuela, 2001; Scahill et al., 2003; Peters, 2006).

There are currently few strategies that have been shown to maintain attentional abilities in older adults. This is problematic as older adults report decreased quality of life (Hoe et al., 2009; Williams and Kemper, 2010) in concert with symptoms of broadly reduced attention and cognition. Listening to music may be one mechanism to help older adults maintain cognition, including attentional performance (Koelsch, 2010, 2014). In an early study, Rauscher et al. (1993) instructed participants to listen to Mozart’s sonata, an instructive relaxation tape, or silence for 10 min before completing a visuospatial task. Their results showed that participants who listened to Mozart performed better on the visuospatial task than the participants in the other two conditions. This effect, which describes better cognitive performance after listening to classical music (Rauscher et al., 1993), was popularized by the media and coined the “Mozart effect.” While Rauscher et al. (1995) replicated their original results in a follow up study, subsequent studies conducted by other research groups, failed to replicate this so called “Mozart effect” (Steele et al., 1997, 1999; McCutcheon, 2000), which led to the development of a new theory: the “arousal-mood” hypothesis (Husain et al., 2002).

The “arousal-mood” hypothesis posits that the tempo and composition mode of music increases arousal and mood, which subsequently increases cognitive performance (Husain et al., 2002). This hypothesis arose in part from research showing that music composed with a fast tempo and in the major mode increases arousal and feelings of happiness, while musical pieces with a slow tempo written in the minor mode decreases arousal and induces feelings of sadness (Balch et al., 1999; Gabrielsson and Lindström, 2001; Thompson et al., 2001; Husain et al., 2002; McConnell and Shore, 2011b). The “arousal-mood” hypothesis was first tested by having participants listen to one of three acoustic conditions: Mozart’s sonata (a happy-sounding, composition with a fast tempo predicted to increase arousal and induce feelings of happiness), Albinoni adagio (a sad-sounding, slow piece predicted to decrease participant arousal and induce feelings of sadness), or 10 min of silence (Thompson et al., 2001). Participants then completed the same visuospatial task used by Rauscher et al. (1993). Arousal level and mood were measured using questionnaires before and after music listening. Thompson et al. (2001) found that participants who listened to Mozart performed better on the visuospatial task than those who listened to Albinoni or silence. The Mozart group also reported increased arousal and happiness compared to the Albinoni group. The authors argued that participants’ cognitive performance improved because listening to Mozart’s sonata increased arousal and induced feelings of happiness (Thompson et al., 2001). Subsequent studies testing this theory have drawn similar conclusions: listening to happy music, written in the major mode with a fast tempo, enhances performance on a variety of visuospatial and processing speed tasks (Thompson et al., 2001; Husain et al., 2002; Schellenberg and Hallam, 2005; Schellenberg et al., 2007; Schellenberg, 2012), working memory tasks (Mammarella et al., 2007; Chew et al., 2016; Palmiero et al., 2016), and attention tasks (Marti-Marca et al., 2020; Kiss and Linnell, 2021), including investigations of executive control (Finucane et al., 2010; Bruyneel et al., 2013; Fernandez et al., 2019). It is possible that the fast tempo and major mode of happy music increases attention, particularly executive control by not only increasing arousal and mood (Balch et al., 1999; Gabrielsson and Lindström, 2001; Thompson et al., 2001; Husain et al., 2002), but also by engaging the brain regions which support attention (Fernandez et al., 2019). Happy music may increase alerting attention similarly to executive control since both subsystems are associated with similar regions in the frontal cortex (Fan et al., 2005; Roberts and Hall, 2008; Anderson et al., 2010; Petersen and Posner, 2012; Rinne et al., 2013).

The purpose of this study was to investigate how listening to happy music, written in the major mode with a fast tempo, impacts alerting, orienting, and executive control attention in middle and older-aged adults. We specifically recruited non-musicians to test for acute changes in attention, as such effects in musicians would be less detectable because long-term musical training preserves cognition, including executive control (Román-Caballero et al., 2018; Medina and Barraza, 2019). The attentional subsystems were assessed using the ANT before (time one) and after (time two) participants listened to happy music (major mode, fast tempo), sad music (minor mode, slow tempo), or no music (i.e., silence) for 10 min. Participants listened to music prior to completing the task as the irrelevant sound effect consistently demonstrates that listening to background music impairs task performance (Ransdell and Gilroy, 2001; Cassidy and MacDonald, 2007; Rowe et al., 2007; Jiang et al., 2011; Rey, 2012; Gonzalez and Aiello, 2019; Cloutier et al., 2020). We hypothesized that listening to happy music, but not sad or no music, would increase alerting and executive control from time one to time two. No changes in orienting attention were expected from time one to time two for any group. Alerting and executive control are known to interact (Callejas et al., 2004; Fan et al., 2009; Gamboz et al., 2010; Ishigami and Klein, 2010; McConnell and Shore, 2011a; Weinbach and Henik, 2012). Therefore, we expected a time x congruency x cue x group interaction such that the alerting effect within incongruent trials would not be present at time one for any group and would only be present at time two for the happy music listening group. We did not expect happy music to impact congruent or neutral trials since the alerting effect should be present at time one for both trial types.

## 2. Materials and methods

### 2.1. Participants

Fifty-nine neurotypical adults were recruited for this study. Two participants were excluded for failing to follow instructions (*n* = 2), resulting in 57 participants (48 females) who ranged in age from 50 to 84 years (*M* = 61.09, SD = 7.16) being included in the final sample. All participants spoke English and had no self-reported history of psychiatric or neurological disease and reported normal or corrected to normal vision. Participants were assigned to one of three experimental groups at the time of study enrollment. The three groups did not differ by age, gender, education, hearing (pure tone average of 500–4,000 Hz in both ears), years of musical training, testing time, basic cognitive abilities (assessed *via* the *Montreal Cognitive Assessment MoCA*; Nasreddine et al., 2005), caffeine intake, sleep, or exercise (Table 1). Participants were monetarily compensated for their time. Midwestern University’s Institutional Review Board approved all procedures, and all participants provided informed consent for the study.

**Table 1:** Means and standard deviations (in parentheses) for the demographic and study specific comparisons between the happy (*n* = 19), sad (*n* = 20), and no music (*n* = 18) listening groups.

	Happy music	Sad music	No music	Statistic
Age	62.37 (8.06)	59.70 (7.01)	61.28 (6.39)	*F* (2,54) = 0.68, *p* = 0.51
Gender	0.84 (0.38)	0.85 (0.37)	0.89 (0.32)	*F* (2,54) = 0.01, *p* = 0.99
Education (years)	16.84 (4.10)	15.90 (2.57)	15.56 (4.13)	*F* (2,54) = 0.62, *p* = 0.54
MoCA	26.26 (2.02)	26.90 (2.38)	26.11 (1.64)	*F* (2,54) = 0.81, *p* = 0.45
Pure tone average	19.38 (7.36)	16.22 (7.88)	20.14 (8.10)	*F* (2,54) = 1.40, *p* = 0.26
Musical training (years)	1.74 (2.88)	1.35 (2.66)	5.56 (14.06)	*F* (2,54) = 1.48, *p* = 0.24
Participation time (military)	1197.63 (246.10)	1314.75 (252.26)	1191.11 (276.72)	*F* (2,54) = 1.41, *p* = 0.25
Sleep (hours)	7.24 (1.10)	6.73 (1.01)	7.19 (0.86)	*F* (2,54) = 1.59, *p* = 0.21
Caffeine (ounces)	7.32 (0.81)	9.00 (6.34)	5.56 (7.66)	*F* (2,54) =1.03, *p* = 0.36
Exercise (minutes)	10.26 (19.89)	6.00 (12.31)	10.00 (30.87)	*F* (2,54) = 0.23, *p* = 0.80

### 2.2. Experimental procedures

**Figure 1:**
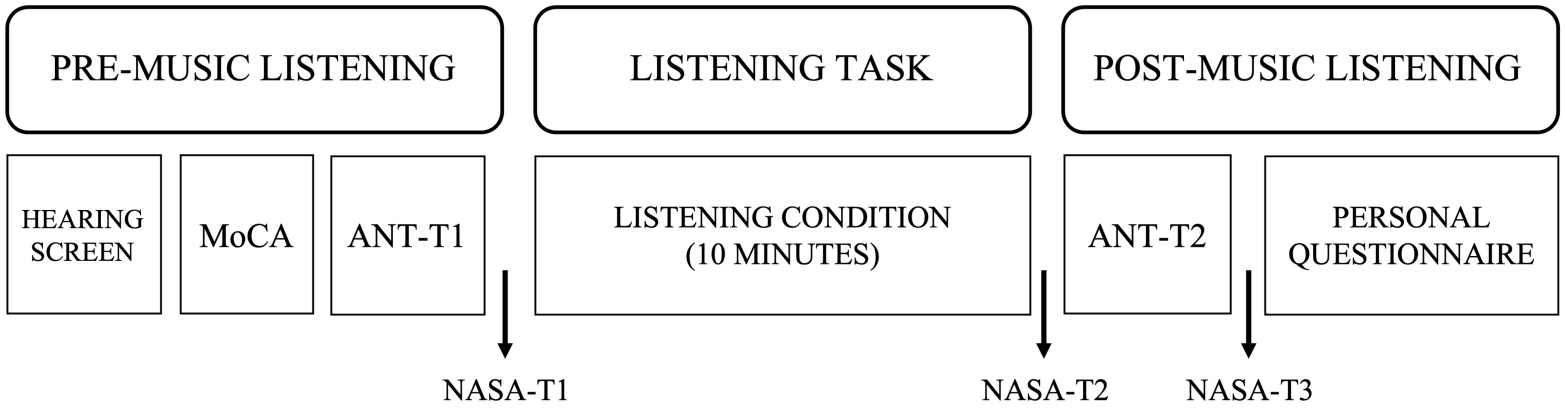
Visual representation of the experimental design.

Participants were assigned to one of three experimental groups upon study enrollment. Group assignment was initially randomized. Toward the end of data collection, a minimization approach was adopted to ensure the groups were comparable on variables of interest such as age, gender, and testing time (Saint-Mont, 2015). Two groups required participants to listen to music, happy music (*n* = 19) or sad music (*n* = 20), and the third group was a no music listening (or silent) control condition (*n* = 18). All groups completed the same study procedures (see Figure 1). Participants’ attention was measured using the Attention Network Test (ANT) before (ANT-T1) and after (ANT-T2) listening to music or silence for 10 min. The ANT and listening tasks were programmed in E-prime 3.0 (Psychology Software Tools, Pittsburgh, PA) on an EyeLink 1,000 Plus eye-tracker (SR Research Ltd., Ottawa, ON, Canada). These additional physiological measures of attentional effort were collected as part of a larger project aiming to answer complementary, yet different questions about the time-course of attention following music listening; as such, analyzes of these data are beyond the scope of this paper.

Participants’ cognitive status and hearing were screened using the *MoCA* and pure tone averages, respectively. Participants additionally completed a questionnaire which asked about demographic information, music familiarity, caffeine intake, sleep, exercise, and general health. The NASA Task Load Index (TLX; Hart and Staveland, 1988) was administered at three timepoints throughout the experiment to assess self-perceived effort after the ANT-T1 (NASA-T1), after listening to music or silence for 10 min (NASA-T2), and after completing the ANT-T2 (NASA-T3).

### 2.3. Music stimuli

Two non-lyrical, classical music pieces were selected based on work done by Trost et al. (2012). These authors had participants rate various musical pieces based on evoked emotions using the Geneva Emotional Music Scale (Zentner et al., 2008), and arousal and valence using a series of multidimensional questionnaires on adjectives of emotion. The “happy music” piece we selected was “Violin Sonata in F Major, 3. Assai vivace” by Felix Mendelssohn. This piece is written in the major mode with a fast tempo and is associated with joyfulness, as well as feelings of high arousal and high valence (Trost et al., 2012). The “sad music” piece we selected was “String Quartet, No. 8 in C Minor, Op. 110, 1. Largo” by Dimitri Shostakovich. This piece is written in the minor mode with a slow tempo and has been associated with sadness and low arousal and low valence (Trost et al., 2012). In addition to evoking opposing emotions, these two compositions were equally familiar to participants (Trost et al., 2012). The original duration of the happy and sad music pieces was 5:14 and 4:53 min, respectively. Each musical piece was presented on a loop through noise canceling headphones for a total of 10 min at a sound level of 60 decibels. The no music listening groups’ (or control groups’) listening procedure was identical to the happy and sad music listening groups’, except they listened to silence for 10 min rather than music. For the two music listening groups, participants rated their perceived emotion following music listening using a visual analog scale anchored at “0″ (sadness) and “100″ (happiness); a rating of “50″ indicated neutral emotion. Participants also rated their familiarity with the musical composition they listened to using a questionnaire which asked if they were unfamiliar (0), somewhat familiar (1), or very familiar (2) with the music.

### 2.4. Attention network task

**Figure 2:**
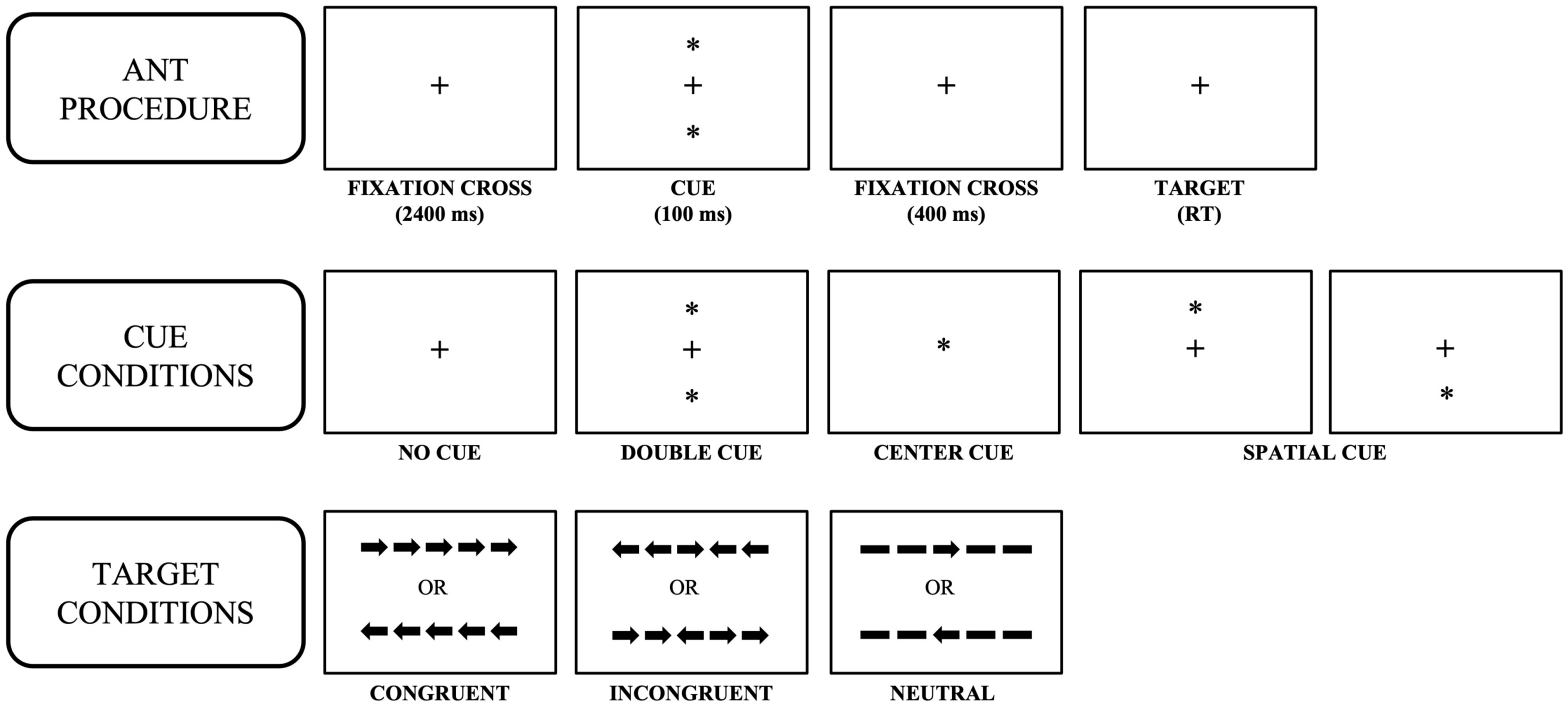
The Attention Network Test (ANT) procedure, cues, and targets.

Participants completed a modified version of the ANT developed by Fan et al., 2002 (Figure 2). Each trial began with a fixation cross for 2,400 milliseconds. Following the offset of the fixation cross, a visual cue was presented for 100 milliseconds. Visual cue conditions were as follows: (1) spatial cue (single asterisk presented either above or below the fixation cross; spatial cues predicted the location of the flanker task 75% of the time (Fan et al., 2009); these will be referred to as valid spatial cues), (2) double cues (simultaneous presentation of one asterisk above the fixation cross and one asterisk below the fixation cross), (3) center cue (single asterisk presented in the center of the screen), and (4) no cue (no cueing was provided; i.e., no offset of the fixation cross). Following the offset of the cue, the fixation cross was presented on the screen for 400 milliseconds after which time participants were presented with the flanker stimuli either above or below the fixation cross.

The flanker stimuli consisted of five arrows pointing left or right. A congruent trial occurred when the center arrow was flanked by arrows pointing in the same direction (i.e., all arrows pointing to the left or right). An incongruent trial occurred when the center arrow pointed in the opposite direction of the flanking arrows (e.g., the center arrow points left, and the flanking arrows point right). A neutral trial occurred when the center arrow was flanked by dashed lines (two on each side). Accuracy and reaction time were collected *via* keyboard button press. Participants were instructed to be fast and accurate and press the left arrow key if the center arrow pointed to the left and the right arrow key if the center arrow pointed to the right. Participants completed a total of 156 trials across two blocks (78 trials x 2 blocks = 156 total trials) at each timepoint (ANT-T1, ANT-T2). For each ANT, all cues were presented equally (36 trials) across both blocks, except for the invalid spatial cue which was presented on 12 trials across both blocks. All flanker targets were presented equally (52 trials per condition) across both ANT blocks. This combination of cues and targets allowed us to calculate three measures of attention using this single task: alerting (no cue-double cued trials), orienting (center cued-valid spatially cued trials), and executive control (incongruent-neutral trials). These difference scores are interpreted as follows: large, positive difference scores equal better alerting and orienting and difference scores closer to zero equal better executive control. Trial presentation was randomized for each participant. Verbal and written instructions, examples of all stimuli, and 10 practice trials preceded the start of the experiment.

### 2.5. NASA task load index (NASA-TLX)

The NASA-TLX (Hart and Staveland, 1988) was used to measure participant’s perceived mental workload, attentional allocation, and physical effort at three timepoints throughout the experiment (NASA-T1, NASA-T2, NASA-T3; Figure 1). The NASA-TLX consists of six questions and was administered as originally published. Participants rated their perceived effort using a visual analog scale anchored at “0” (very low effort) and “100” (very high effort). Participant’s average raw score across all six questions was analyzed to ensure self-perceived effort was equivalent across groups as several studies indicate that effort affects cognitive task performance (Inzlicht et al., 2018).

### 2.6. Statistical analysis

#### 2.6.1. Perceptions of music listening condition

All data were analyzed in SPSS Version 27 (IBM, 2020). Independent samples *t-*tests were used to compare how participants in the happy and sad music listening groups rated the emotions evoked by each composition and their familiarity with the composition. Changes in participants self-reported effort across the experiment using the NASA-TLX were analyzed using a mixed ANOVA with three levels of group (happy music, sad music, no music listening control group) and three levels of time (NASA-T1, NASA-T2, and NASA-T3); the dependent variable was the average raw score across all six questions.

#### 2.6.2. Changes in attention after music listening

Accuracy and reaction time (RT; measured in milliseconds) were collected for each ANT trial. Reaction time is our behavioral measure of interest as it is widely accepted that older adults demonstrate a speed-accuracy tradeoff where they favor accuracy over speed (see Heitz, 2014 for a review). Only reaction times associated with correct responses were included in the analyzes (0.6% of trials were removed for incorrect responses). We first used paired sample *t-*tests to test our hypothesis that listening to happy music would increase alerting and executive control attention from time one to time two, but sad and no music listening would not. We used reaction time difference scores to calculate the alerting (no cue–double cue), orienting (center cue–valid spatial cue), and executive control (incongruent–neutral trials) effects at time one and time two for each group. Reaction time difference scores allowed us to isolate any music-induced changes to alerting, orienting, and executive control from individual variability in general processing speed, motor response speeds, and practice effects.

A 3×2×3×4 mixed ANOVA with three levels of group (happy music, sad music, no music listening control group), two levels of time (ANT-T1, ANT-T2), three levels of congruency (congruent, incongruent, neutral), and four cue types (no cue, double cue, center cue, and valid spatial cue^1^) was conducted to test our hypothesis that group, time, cue, and congruency would interact such that happy music would change the efficiency of the alerting subsystem from time one to time two within incongruent trials only. The Greenhouse–Geisser correction was used to correct for violations to sphericity. Post-hoc comparisons were corrected for multiple comparisons using the Benjamini-Hochberg (BH) false discovery rate (FDR; Benjamini and Hochberg, 1995).

## 3. Results

### 3.1. Perceptions of music listening condition

Participants’ familiarity with each musical composition did not differ [*t*(37) = 1.13, *p* = 0.27; Happy Music: *M* = 0.79, SD = 0.71; Sad Music: *M* = 0.55, SD = 0.61]. Consistent with expectations, there was a significant difference in how participants rated the emotions tied to each musical piece [*t*(37) = 6.39, *p* < 0.001]: participants rated the happy music (*M* = 79.74, SD = 12.19) higher on the visual analog scale than the sad music (*M* = 44.75, SD = 20.68). While there was no effect of group on the NASA-TLX [*F*(2, 53) = 0.45, *p* = 0.64] and group did not interact with time [*F*(4, 106) = 0.87, *p* = 0.49], all participants rated the music listening phase as the most cognitively taxing [*F*(1.46, 77.24) = 22.74, *p* < 0.001; NASA-T1: *M* = 15.94, SD = 12.39; NASA-T2: *M* = 27.54, SD = 19.44; NASA-T3: *M* = 19.63, SD = 15.39].

### 3.2. Changes in attention after music listening

#### 3.2.1. Paired sample *t-*tests

**Figure 3:**
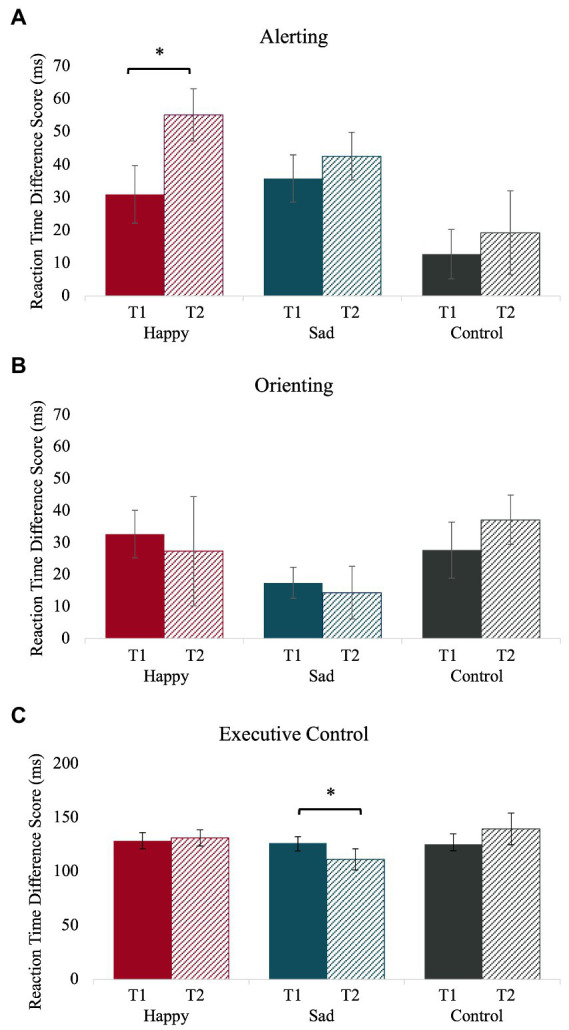
Changes in alerting **(A)** reaction time difference score for no cue–double cue, orienting **(B)** reaction time difference score for center cue–valid spatial cue, and executive control **(C)** reaction time difference score for incongruent–neutral trials from time one (T1) to time two (T2) within each group. Error bars represent ± 1 standard error. Alerting improved from time one to time two for the happy music group only. Executive control improved from time one to time two for the sad music group only.

**Figure 4:**
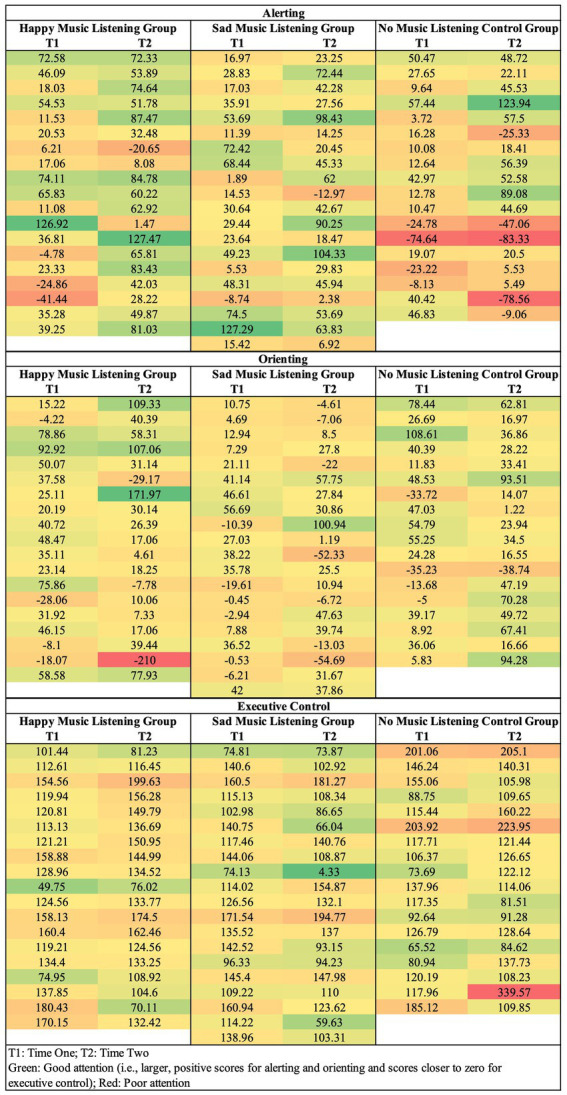
Individual variation in how alerting (reaction time difference score for no cue–double cue), orienting (reaction time difference score for center cue–valid spatial cue), and executive control (reaction time difference score for incongruent–neutral trials) change from time one to time two for each participant. Green shading represents better attention and red shading poorer attention. The individual data depicts a similar pattern as the whole-group analysis: alerting attention predominately changes in response to happy music and executive control in response to sad music.

The means and standard deviations for the ANT separated by group and time are reported in Table 2. Reaction time difference scores for each group on the ANT-T1 (time one) and the ANT-T2 (time two) are plotted in Figure 3. Individual variability in how alerting, orienting, and executive control change from time one to time two are depicted in Figure 4. As expected, the happy music listening group was more alert at time two than time one [i.e., they had larger reaction time difference scores at time two compared to time one; *t*(18) = 2.09, *p = 0*.05, *d =* 0.48]. Alertness did not change from time one to time two for the sad [*t*(19) = 0.86, *p = 0*.40, *d =* 0.19] or no music listening groups [*t*(17) = 0.59, *p = 0*.56, *d =* 0.14]. The orienting effect did not change from time one to time two for any group: happy [*t*(18) = 0.33, *p = 0*.74, *d =* 0.08], sad [*t*(19) = 0.31, *p = 0*.76, *d =* 0.07], and no music listening [*t*(17) = 0.91, *p = 0*.38, *d =* 0.21]. Unexpectedly, executive control increased from time one to time two for the sad music listening group [i.e., they had smaller reaction time difference scores at time two than time one; *t*(19) = 2.07, *p = 0*.05, *d =* 0.46]. Executive control did not change from time one to time two for the happy [*t*(18) = 0.32, *p = 0*.75, *d =* 0.07] or no music listening groups [*t*(17) = 0.99, *p = 0*.34, *d =* 0.23].

**Table 2:** The means and standard deviations (in parentheses) for the ANT separated by group and time.

	Cue types					Congruency conditions			
Group	No cue	Double	Center	Valid spatial	Invalid spatial	Congruent	Incongruent	Neutral	All
ANT-T1									
Happy	702.42 (116.55)	671.46 (98.47)	683.82 (104.47)	651.11 (102.31)	708.26 (117.03)	644.24 (95.30)	761.77 (118.55)	633.27 (102.16)	679.76 (103.7)
Sad	657.39 (100.97)	621.58 (92.84)	633.26 (91.95)	615.83 (96.26)	675.36 (118.20)	604.08 (97.35)	714.49 (98.19)	588.21 (91.09)	635.59 (93.94)
Control	673.66 (102.72)	660.90 (101.76)	661.84 (105.17)	634.17 (87.99)	696.87 (140.65)	622.64 (97.60)	742.80 (112.74)	617.65 (95.65)	661.03 (100.32)
All	677.54 (106.71)	650.63 (98.33)	659.14 (100.91)	633.38 (95.27)	693.12 (123.87)	623.33 (96.47)	739.19 (109.74)	612.53 (96.50)	658.35 (99.24)
ANT-T2									
Happy	667.68 (89.37)	612.56 (79.80)	636.93 (87.95)	609.59 (104.24)	642.71 (81.36)	596.08 (82.24)	716.89 (90.59)	585.77 (86.41)	632.91 (83.42)
Sad	638.68 (107.50)	596.11 (94.72)	596.43 (90.35)	582.04 (96.38)	627.55 (98.66)	571.61 (96.10)	677.90 (100.77)	566.72 (92.98)	605.41 (94.68)
Control	647.98 (101.50)	628.70 (110.91)	620.09 (98.83)	582.93 (79.23)	636.40 (88.15)	590.79 (94.58)	707.46 (112.70)	567.96 (82.44)	622.07 (93.48)
All	651.29 (98.82)	611.89 (94.89)	617.40 (92.25)	591.51 (93.38)	635.39 (88.49)	585.82 (90.21)	700.23 (101.13)	573.46 (86.45)	619.84 (89.8)
ANT: collapsed across time									
Happy	685.05 (99.37)	642.01 (87.56)	660.38 (92.00)	630.35 (99.18)	675.49 (91.72)	620.16 (84.16)	739.33 (103.27)	609.52 (91.17)	656.34 (91.68)
Sad	648.04 (97.95)	608.85 (90.32)	614.84 (86.68)	598.94 (92.25)	651.45 (97.76)	587.84 (90.70)	696.19 (95.54)	577.46 (88.25)	620.50 (90.27)
Control	660.82 (95.04)	644.80 (96.72)	640.97 (93.85)	608.55 (76.44)	666.63 (105.99)	606.72 (88.72)	725.13 (103.38)	592.81 (81.43)	641.55 (89.60)
All	664.41 (97.03)	631.26 (91.38)	638.27 (91.15)	612.44 (89.45)	664.26 (97.27)	604.58 (87.41)	719.71 (100.53)	592.99 (86.64)	639.09 (90.16)

#### 3.2.2. Mixed ANOVA

The means and standard deviations for the ANT separated by time, group, congruency, and cue are reported in Table 3. The full mixed ANOVA results are reported in Table 4. There were no group differences between the happy, sad, and no music listening groups [*F*(2, 54) = 0.81, *p* = 0.45]. The main effect of time was significant [*F*(1, 54) = 23.71, *p* < 0.001]: as expected, all participants produced faster responses at time two than time one. The main effect of cue was significant [*F*(2.61, 140.78) = 57.45, *p* < 0.001]: both the alerting (faster responses on double cue than no cue trials) and orienting effects were significant (faster responses on valid spatial cue than center cue trials). The main effect of congruency was also significant [*F*(1.60, 86.63) = 610.66, *p* < 0.001]: participants responded more slowly on incongruent trials than neutral trials (Tables 2, 4). The congruency x cue interaction was significant [*F*(4.81, 259.87) = 8.02, *p* < 0.001]: the alerting effect was significant for congruent and neutral trials, but not the incongruent trials. The orienting effect was significant in all three congruency conditions (Table 4).

**Table 3:** The means and standard deviations (in parentheses) for the ANT separated by time, group, congruency, and cue.

	Congruent trials				Incongruent trials				Neutral trials			
Group	No Cue	Double	Center	Valid spatial	No cue	Double	Center	Valid spatial	No Cue	Double	Center	Valid spatial
ANT-T1												
Happy	670.15 (100.20)	628.38 (85.79)	647.83 (96.71)	611.60 (105.70)	756.14 (105.66)	768.56 (121.68)	780.77 (137.48)	734.14 (123.25)	681.73 (159.11)	618.07 (100.26)	623.77 (96.40)	608.18 (90.55)
Sad	639.75 (101.17)	585.02 (97.86)	595.80 (87.83)	576.68 (102.15)	725.06 (121.26)	706.72 (93.15)	720.50 (100.72)	691.43 (98.32)	608.03 (90.33)	574.65 (94.85)	585.00 (91.37)	579.84 (99.57)
Control	640.04 (106.70)	613.34 (93.09)	613.88 (96.07)	608.88 (100.73)	737.32 (109.15)	768.17 (125.21)	756.65 (133.40)	696.38 (99.11)	644.26 (117.93)	603.13 (92.20)	615.32 (101.39)	598.08 (79.44)
All	649.97 (101.80)	608.42 (92.65)	618.85 (94.38)	598.49 (102.35)	739.29 (111.19)	746.74 (115.51)	752.00 (124.74)	707.23 (107.32)	644.04 (126.78)	598.12 (95.94)	607.50 (96.08)	595.05 (89.77)
ANT-T2												
Happy	641.62 (93.44)	565.55 (80.40)	600.29 (122.64)	575.50 (110.46)	742.90 (110.74)	707.47 (92.03)	727.43 (96.23)	678.41 (98.75)	619.69 (94.52)	565.44 (78.25)	583.76 (94.38)	575.37 (152.64)
Sad	608.10 (116.83)	565.31 (110.98)	549.73 (82.33)	559.23 (95.97)	690.73 (110.61)	680.12 (95.60)	678.49 (98.06)	651.91 (115.03)	617.50 (105.39)	543.95 (86.81)	561.45 (100.25)	535.99 (91.40)
Control	640.12 (129.14)	590.72 (119.17)	588.31 (105.63)	542.72 (77.42)	710.78 (113.59)	725.72 (128.31)	710.22 (118.55)	676.34 (128.69)	593.95 (78.15)	571.19 (114.36)	565.36 (88.21)	535.27 (71.32)
All	629.38 (112.92)	573.41 (103.48)	578.77 (104.93)	559.44 (95.10)	714.45 (111.76)	703.64 (105.74)	704.82 (104.57)	668.46 (113.15)	610.79 (92.89)	559.71 (92.94)	570.12 (93.45)	548.89 (110.57)
ANT: collapsed across time												
Happy	655.89 (90.13)	596.96 (76.64)	624.06 (91.75)	593.55 (103.54)	749.52 (104.89)	738.02 (104.75)	754.10 (113.05)	706.27 (103.62)	650.71 (114.87)	591.76 (86.94)	603.76 (93.82)	591.77 (111.51)
Sad	623.92 (97.43)	575.16 (100.68)	572.77 (79.86)	567.96 (94.68)	707.90 (109.82)	693.42 (89.62)	699.49 (94.92)	671.67 (101.54)	612.77 (92.87)	559.30 (86.63)	573.22 (88.92)	557.91 (89.74)
Control	640.08 (114.34)	602.03 (92.65)	601.09 (92.92)	575.80 (72.67)	724.05 (97.66)	746.94 (114.11)	733.44 (115.67)	686.36 (102.27)	619.11 (87.07)	587.16 (95.31)	590.34 (85.51)	566.68 (69.04)
All	639.68 (99.89)	590.91 (89.85)	598.81 (89.17)	578.96 (90.60)	726.87 (104.06)	725.19 (103.79)	728.41 (108.43)	687.84 (101.65)	627.42 (98.75)	578.92 (89.16)	588.81 (89.14)	571.97 (91.59)

**Table 4:** The full mixed ANOVA model results for reaction time across both ANT time points.

Effect, interaction, or contrast	Statistic
Group	*F*(2, 54) = 0.81, *p = 0*.45, η_p_^2^ = 0.03
Time	*F*(1, 54) = 23.71, *p* < 0.001^*^, η_p_^2^ = 0.31
Time x group	*F*(2, 54) = 0.41, *p* = 0.67, η_p_^2^ = 0.02
Congruency	*F*(1.60, 86.63) = 610.66, *p* < 0.001*, η_p_^2^ = 0.91
Cue	*F*(2.61, 140.78) = 57.45, *p* < 0.001*, η_p_^2^ = 0.52
Congruency x group	*F*(4, 108) = 0.73, *p* = 0.58, η_p_^2^ = 0.03
Cue x group^***^	*F*(6, 162) = 2.33, *p* = 0.035^*^, η_p_^2^ = 0.08
Time x congruency	*F*(2, 108) = 0.57, *p* = 0.57, η_p_^2^ = 0.10
Time x congruency x group	*F*(4, 108) = 1.36, *p* = 0.25, η_p_^2^ = 0.05
Time x cue	*F*(3, 162) = 2.69, *p* = 0.048^*^, 0.05
Time x cue x group	*F*(6, 162) = 1.01, *p* = 0.42, η_p_^2^ = 0.04
Congruency x cue	*F*(4.81, 259.87) = 8.02, *p* < 0.001*, η_p_^2^ = 0.13
Congruency x cue x group	*F*(12, 324) = 0.42, *p* = 0.96, η_p_^2^ = 0.02
Time x congruency x cue	*F*(4.44, 240.04) = 0.43, *p* = 0.81, η_p_^2^ = 0.01
Time x congruency x cue x group	*F*(12, 324) = 2.35, *p* = 0.007^*^, η_p_^2^ = 0.08
Pairwise comparisons for the main effect of congruency	
Executive control (incongruent vs. neutral)	*t*(56) = 27.80, FDR *p* = 0.001^**^, *d =* 3.68
Incongruent vs. congruent	*t*(56) = 27.66, FDR *p* = 0.001^**^, *d =* 3.66
Congruent vs. neutral	*t*(56) = 4.25, FDR *p* = 0.001^**^, *d =* 0.56
Pairwise comparisons for the main effect of cue	
Alerting (no cue vs. double cue)	*t*(56) = 7.75, FDR *p* = 0.001^**^, *d =* 1.03
Orienting (center cue vs. valid spatial cue)	*t*(56) = 5.90, FDR *p* = 0.001^**^, *d =* 0.78
No cue vs. center cue	*t*(56) = 6.96, FDR *p* = 0.001^**^, *d =* 0.92
No cue vs. valid cue	*t*(56) = 11.18, FDR *p* = 0.001^**^, *d =* 1.48
Double cue vs. center cue	*t*(56) = 2.17, FDR *p* = 0.034^**^, *d =* 0.29
Double cue vs. valid cue	*t*(56) = 4.61, FDR *p* = 0.001^**^, *d =* 0.61
Pairwise comparisons for the interaction: congruency x cue	
Congruent: alerting	*t*(56) = 7.31, FDR *p* = 0.002^**^, *d =* 0.97
Congruent: orienting	*t*(56) = 3.06, FDR *p* = 0.005^**^, *d =* 0.41
Incongruent: alerting	*t*(56) = 0.30, FDR *p* = 0.76, *d =* 0.04
Incongruent: orienting	*t*(56) = 5.97, FDR *p* = 0.002^**^, *d =* 0.79
Neutral: alerting	*t*(56) = 8.25, FDR *p* = 0.002^**^, *d =* 1.09
Neutral: orienting	*t*(56) = 2.16, FDR *p* = 0.042^**^, *d =* 0.27

*Significant at *p* < 0.05; **Significant at FDR *p* < 0.05; ***All post-hoc comparisons were non-significant at *p* < 0.05. The alerting effect is characterized by faster responses on double cue compared to no cued trials. The orienting effect is characterized by faster responses on trials with valid spatial cues compared to trials with center cues.

**Figure 5:**
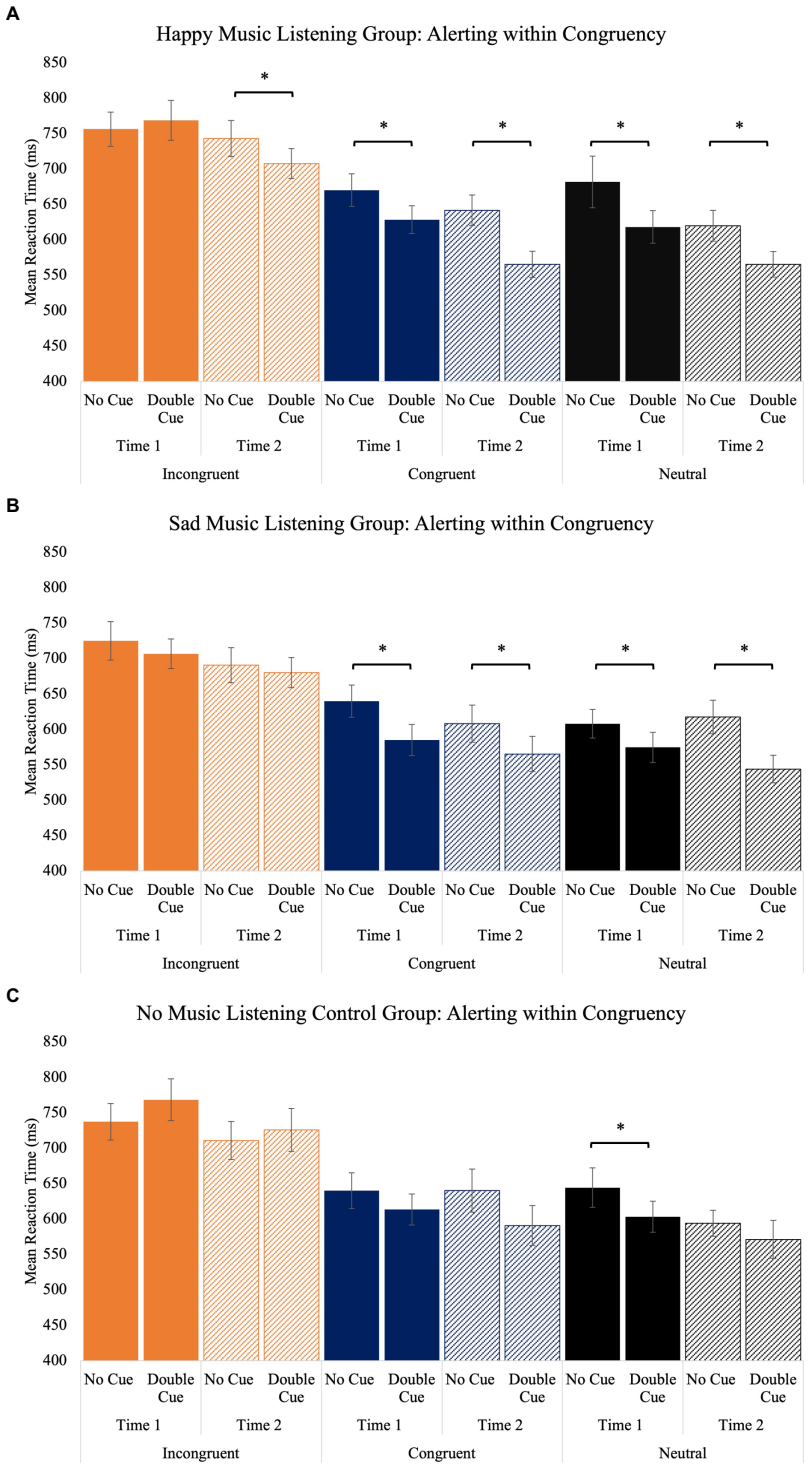
The alerting (no cue vs. double cue) effect separated by congruency (incongruent, congruent, and neutral) for the happy **(A)**, sad **(B)**, and no music **(C)** listening groups. Error bars represent ± 1 standard error. Note, within the control group, there is a significant difference between the no cue and double cue trials within neutral trials at time one. This difference is not marked as significant as it is in the opposite direction of the alerting effect (i.e., no cue trials were faster than double cue trials).

The time x congruency x cue x group interaction was also significant [*F*(12, 273.93) = 2.35, *p* = 0.007]. As expected, no group demonstrated the expected alerting effect (faster responses on double cue than no cue trials) within incongruent trials at time one, and only the happy music listening group demonstrated the alerting effect within incongruent trials at time two. Within congruent and neutral trials, the alerting effect was present at time one and time two for both the happy and sad music groups. For the control group, the alerting effect was observed at time one for neutral trials, but not congruent trials. The alerting effect was not present at time two within congruent or neutral trials for the control group (Table 5; Figure 5). Supplementary Figure S1 depicts individual differences in how alerting changes within incongruent, congruent, and neutral trials from time one to time two for each participant.

**Table 5:** Post-hoc comparisons within reaction time for the group x time x congruency x cue interaction.

Contrast	Statistic
Happy music group	
Time 1: incongruent: alerting	*t*(18) = −1.55, FDR *p* = 0.19, *d =* 0.36
Time 2: incongruent: alerting	*t*(18) = 2.76, FDR *p* = 0.022^*^, *d =* 0.63
Time 1: congruent: alerting	*t*(18) = 3.52, FDR *p* = 0.006^*^, *d =* 0.81
Time 2: congruent: alerting	*t*(18) = 6.11, FDR *p* = 0.006^*^, *d =* 1.40
Time 1: neutral: alerting	*t*(18) = 3.52, FDR *p* = 0.006^*^, *d =* 0.81
Time 2: neutral: alerting	*t*(18) = 3.77, FDR *p* = 0.006^*^, *d =* 0.87
Time 1: incongruent: orienting	*t*(18) = 2.60, FDR *p* = 0.027^*^, *d =* 0.60
Time 2: incongruent: orienting	*t*(18) = 3.33, FDR *p* = 0.008^*^, *d =* 0.76
Time 1: congruent: orienting	*t*(18) = 3.42, FDR *p* = 0.007^*^, *d =* 0.78
Time 2: congruent: orienting	*t*(18) = 0.86, FDR *p* = 0.44, *d =* 0.20
Time 1: neutral: orienting	*t*(18) = 1.18, FDR *p* = 0.30, *d =* 0.27
Time 2: neutral: orienting	*t*(18) = 0.25, FDR *p* = 0.80, *d =* 0.06
Sad music group	
Time 1: incongruent: alerting	*t*(19) = 1.49, FDR *p* = 0.20, *d =* 0.33
Time 2: incongruent: alerting	*t*(19) = 1.22, FDR *p* = 0.29, *d =* 0.27
Time 1: congruent: alerting	*t*(19) = 4.73, FDR *p* = 0.004^*^, *d =* 1.06
Time 2: congruent: alerting	*t*(19) = 3.47, FDR *p* = 0.009^*^, *d =* 0.78
Time 1: neutral: alerting	*t*(19) = 6.04, FDR *p* = 0.006^*^, *d =* 1.35
Time 2: neutral: alerting	*t*(19) = 7.98, FDR *p* = 0.006^*^, *d =* 1.78
Time 1: incongruent: orienting	*t*(19) = 3.09, FDR *p* = 0.01^*^, *d =* 0.69
Time 2: incongruent: orienting	*t*(19) = 1.92, FDR *p* = 0.11, *d =* 0.43
Time 1: congruent: orienting	*t*(19) = 2.69, FDR *p* = 0.03^*^, *d =* 0.60
Time 2: congruent: orienting	*t*(19) = −0.86, FDR *p* = 0.44, *d =* 0.19
Time 1: neutral: orienting	*t*(19) = 0.55, FDR *p* = 0.59, *d =* 0.12
Time 2: neutral: orienting	*t*(19) = 2.08, FDR *p* = 0.09, *d =* 0.47
No music group	
Time 1: incongruent: alerting^**^	*t*(17) = −2.95, FDR *p* = 0.03^*^, *d =* 0.70
Time 2: incongruent: alerting	*t*(17) = −0.99, FDR *p* = 0.37, *d =* 0.23
Time 1: congruent: alerting	*t*(17) = 1.99, FDR *p* = 0.10, *d =* 0.47
Time 2: congruent: alerting	*t*(17) = 2.30, FDR *p* = 0.06, *d =* 0.54
Time 1: neutral: alerting	*t*(17) = 2.51, FDR *p* = 0.048^*^, *d =* 0.59
Time 2: neutral: alerting	*t*(17) = 1.27, FDR *p* = 0.26, *d =* 0.30
Time 1: incongruent: orienting	*t*(17) = 2.96, FDR *p* = 0.03^*^, *d =* 0.70
Time 2: incongruent: orienting	*t*(17) = 1.51, FDR *p* = 0.20, *d =* 0.36
Time 1: congruent: orienting	*t*(17) = 0.41, FDR *p* = 0.69, *d =* 0.10
Time 2: congruent: orienting	*t*(17) = 3.47, FDR *p* = 0.02^*^, *d =* 0.82
Time 1: neutral: orienting	*t*(17) = 1.87, FDR *p* = 0.12, *d =* 0.44
Time 2: neutral: orienting	*t*(17) = 3.92, FDR *p* = 0.01^*^, *d =* 0.93

*Significant at FDR *p* < 0.05. **There is a significant difference between the no cue and double cue trials, but in the opposite direction of the alerting effect (i.e., no cue trials were faster than double cue trials). The alerting effect is faster responses on double cue compared to no cued trials. The orienting effect is faster responses on trials with valid spatial cues compared to trials with center cues.

**Figure 6:**
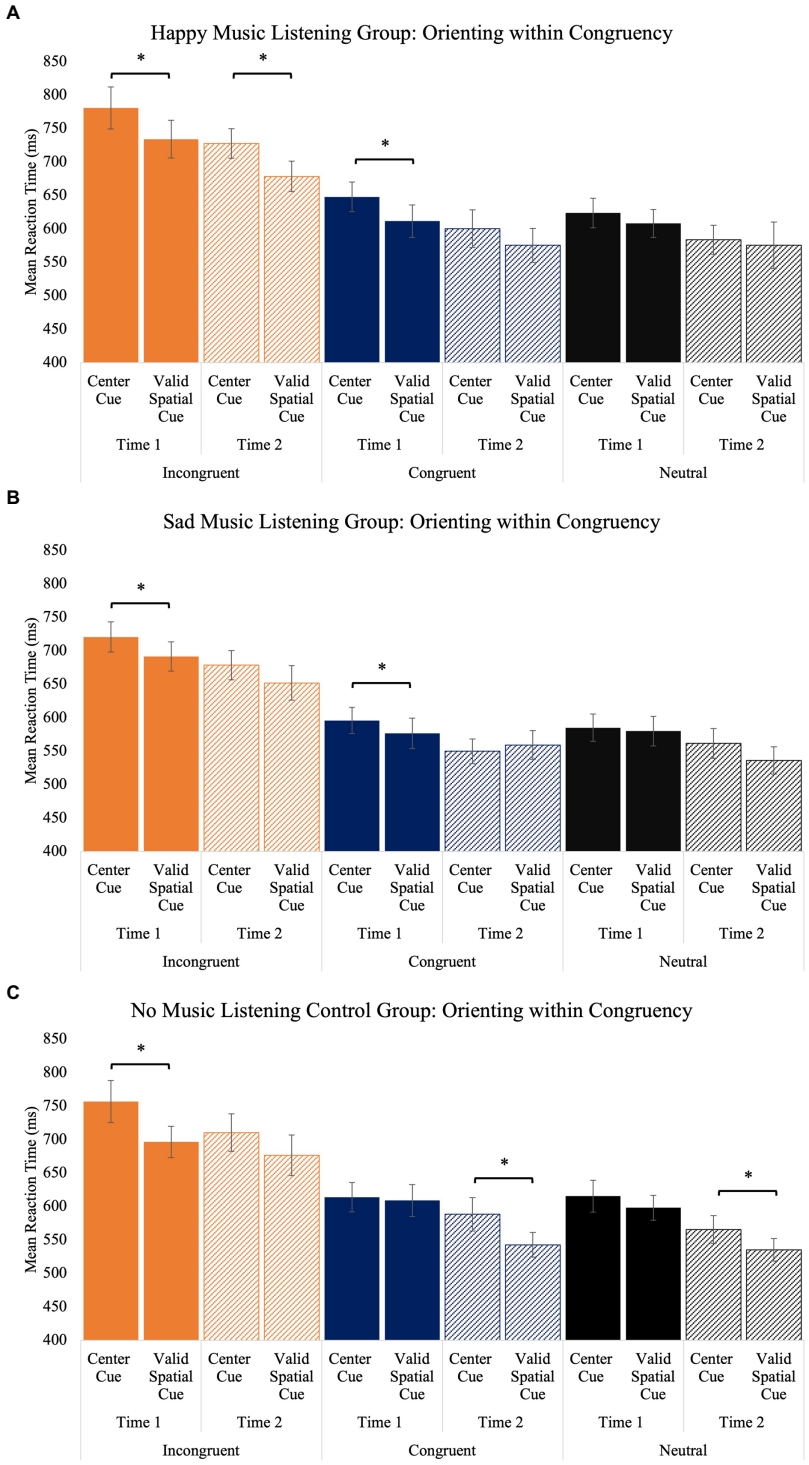
The orienting (center cue vs. valid spatial cue) effect separated by congruency (incongruent, congruent, and neutral) for the happy **(A)**, sad **(B)**, and no music **(C)** listening groups. Error bars represent ± 1 standard error.

We also observed the orienting effect (faster responses on valid spatial cue than center cue trials) to interact with group, time, and congruency. Within incongruent trials, all groups demonstrated the orienting effect at time one, but this effect was only maintained at time two for the happy music listening group. Within congruent trials, the happy and sad music groups showed the orienting effect at time one, but not at time two. The control group did not demonstrate the orienting effect within congruent trials at time one but did at time two. The orienting effect was not observed within any group at time one within neutral trials, and only the control group demonstrated the orienting effect at time two (Table 5; Figure 6). Supplementary Figure S2 depicts individual differences in how orienting changes within incongruent, congruent, and neutral trials from time one to time two for each participant.

## 4. Discussion

It is well-established that attention, specifically alerting and executive control, decline with increasing age (Fernandez-Duque and Black, 2006; Jennings et al., 2007; Gamboz et al., 2010; Mahoney et al., 2010; Zhou et al., 2011; Knight and Mather, 2013; Kaufman et al., 2016; Williams et al., 2016), likely due to atrophy in the prefrontal cortex as part of the normal aging process (Peters, 2006; Glisky, 2007; Zanto and Gazzaley, 2019). However, few studies have investigated strategies to maintain attention in older adults. Listening to happy music may be a potential avenue as past research shows that music written in the major mode with a fast tempo increases attentional performance in older adults (Fernandez et al., 2019; Marti-Marca et al., 2020). The present study sought to investigate how listening to happy music (major mode, fast tempo), compared to sad music (minor mode, slow tempo) or no music, acutely impacts alerting, orienting, and executive control attention in older adults. To this end, alerting and executive control improved after listening to happy and sad music, respectively. These results are discussed in their respective sections below.

### 4.1. Alerting and executive control interact before music listening

Consistent with previous literature (Jennings et al., 2007; Gamboz et al., 2010; Zhou et al., 2011), the mixed ANOVA revealed that all participants demonstrated the expected alerting (i.e., faster reaction times on double cue versus no cue trials), orienting (i.e., faster reaction times on valid spatial cue compared to center cued trials), and executive control effects (i.e., slower reaction times on incongruent trials than neutral trials) collapsed across time. The congruency x cue interaction was also significant across time: the alerting effect was present when the flanker task was congruent or neutral, but not when it was incongruent. This finding aligns with previous work and suggests that the attention networks are not entirely independent (Callejas et al., 2004; Fan et al., 2009; Gamboz et al., 2010; Ishigami and Klein, 2010; McConnell and Shore, 2011a; Weinbach and Henik, 2012).

This diminished alerting effect within incongruent trials may be attributed to alerting and executive control utilizing partially shared resources (Fan et al., 2005; Roberts and Hall, 2008; Anderson et al., 2010; Petersen and Posner, 2012; Rinne et al., 2013), as well as the overall limited capacity of attention (Kahneman, 1973). A warning cue plus an incongruent trial may cause older adults to exceed their resource capacity as the warning cue uses more attention resources than no cue. This may subsequently result in reduced performance, or longer response times on the trials in which a warning cue precedes an incongruent trial. The speed-accuracy tradeoff may also contribute to the diminished alerting effect within incongruent trials. Older adults are known to favor accuracy over speed, and this effect is exacerbated on harder tasks (Heitz, 2014). The speed-accuracy tradeoff may specifically affect incongruent trials in that no matter the type of cueing provided, older adults take the necessary time to accurately complete the task, resulting in similar response times across all incongruent trials.

### 4.2. Music interacts with attention

#### 4.2.1. Alerting

Only one study to our knowledge has investigated the impact of music listening prior to completing the ANT. Their results showed that the alerting effect was similar for younger adults who listened to happy (major mode, fast tempo) and sad music (minor mode, slow tempo; McConnell and Shore, 2011b). Our results expand upon this work to show that in older adults, alerting attention increases in response to happy music listening, but not after listening to sad music or no music. Furthermore, when we focus on incongruent trials, the trials most challenging for older adults (Mutter et al., 2005; Ward et al., 2021), we show that the alerting effect was not significant for the happy music group at time one, but was at time two. This same pattern within incongruent trials was not observed for the sad and no music listening groups. These findings correspond to the arousal-mood hypothesis, which posits that listening to non-lyrical music written in the major mode with a fast tempo, induces positive mood and increases levels of arousal more so than sad music or music written in the minor mode with a slow tempo (Balch et al., 1999; Gabrielsson and Lindström, 2001; Thompson et al., 2001; Husain et al., 2002; McConnell and Shore, 2011b). These increases in mood and arousal following happy music listening subsequently result in superior task performance (Steele, 2000; Thompson et al., 2001; Husain et al., 2002; Schellenberg and Hallam, 2005; Schellenberg et al., 2007; Schellenberg, 2012).

In addition to increasing mood and arousal levels, happy music may also improve alerting attention by stimulating its supporting neural resources. Fernandez et al. (2019) found that older adults who listened to happy background music had better alerting attention than those who listened to sad music. They further observed increased activation in the prefrontal cortex for happy music only. Increased activation in the prefrontal cortex during happy music listening was attributed to executive control (Fernandez et al., 2019). However, it could also be associated with alerting attention as fMRI has a relatively poor temporal resolution (Glover, 2011; Loued-Khenissi et al., 2019; Puttaert et al., 2020), which may make it difficult to parse the alerting subsystem from the executive control subsystem given the short interstimulus period between the cue offset and the target onset (i.e., 400 milliseconds). Regardless, music listening activates areas of the brain associated with attention (Mitterschiffthaler et al., 2007; Brattico et al., 2011). Music listening also promotes neural entrainment, specifically in response to musical features such as rhythm and pitch (Nozaradan et al., 2011, 2012; Doelling and Poeppel, 2015; Wollman et al., 2020). Together these findings suggest a potential therapeutic benefit to happy music listening in the short-term, and further show that in some instances happy music may be able to reinstate a previously absent or diminished alert state in older adults. However, additional work is needed to confirm and extend these findings using a more diverse group of participants.

#### 4.2.2. Orienting

The paired samples *t-*tests indicate that the orienting effect did not change in response to music listening. However, further investigation of the orienting effect within the challenging incongruent trials indicates that the orienting effect was significant at time one for all groups, however, was only significant in the happy music listening group at time two. This finding, which indicates that happy music may be modulating orienting attention contrasts with the previous literature showing that orienting attention does not change in response to happy or sad music (Jiang et al., 2011; McConnell and Shore, 2011b; Fernandez et al., 2019). Yet, the majority of research investigating the impact of music listening prior to task completion has utilized visuospatial tasks (Rauscher et al., 1993, 1995; Thompson et al., 2001; Husain et al., 2002; Schellenberg and Hallam, 2005; Schellenberg et al., 2007; Schellenberg, 2012), and the orienting cue provides relevant information about the visuospatial location of the flanker task. Music listening prior to ANT performance may therefore increase participants’ ability to effectively respond to both valid spatial and centrally cued trials, possibly because of increased alertness (Fan et al., 2002). However, future work is needed to explore this hypothesis as alerting and orienting did not correlate (*r*(57) = 0.03, *p =* 0.81), nor interact in this study or others (Fan et al., 2002; Yin et al., 2012; Spagna et al., 2015; Aminihajibashi et al., 2020).

#### 4.2.3. Executive control

The arousal-mood hypothesis suggests that executive control performance should improve after listening to happy music (Thompson et al., 2001; Husain et al., 2002). Older adults have also been shown to perform better on the executive control aspect of the ANT when they listened to happy music compared to sad or no music (Fernandez et al., 2019). Therefore, our finding that executive control attention improved after listening to sad music was unexpected, but does align with work showing that sad music, or music written in the minor mode, can increase executive control attention more so than happy music, or music written in the major mode, particularly when arousal levels are high (McConnell and Shore, 2011b). Listening to sad music may increase executive control attention more so than listening to happy music because sad music focuses attention (Jiang et al., 2011) while happy music broadens attention (Rowe et al., 2007). This broadening of attention subsequently impairs inhibitory control (Rowe et al., 2007). Thus, sad music may increase executive control by narrowing the focus of attention, such that participants are more locally attentive to the information they are cued to attend to rather than globally attentive to all aspects of the flanker task, including the distractor information within the incongruent trials (Rowe et al., 2007; Jiang et al., 2011; McConnell and Shore, 2011b). Sad music may also prime participants to internally focus their attention (Jiang et al., 2011; Taruffi et al., 2017), resulting in improved conflict resolution (Lippelt et al., 2014). However, future work is needed to confirm this relationship between sad music listening and executive control performance as group did not interact with congruency or time in the mixed ANOVA.

### 4.3. Musical considerations and future directions

Overall, this study demonstrates that listening to music increases attentional performance, particularly alerting and executive control. This conclusion may only apply to females, who comprised most of our sample. The majority of research indicates that attention does not differ between females and males (Mahoney et al., 2010; Zhou et al., 2011; McDonough et al., 2019). However, males and females perceive emotion differently (Aljanaki et al., 2016; Cloutier et al., 2020), with females reporting higher levels of happiness than males (Cloutier et al., 2020). It is therefore possible that our predominately female sample contributed to the sad music listening group rating their musical composition more neutrally (44.8/100) than what would be expected based on the arousal-mood hypothesis (Husain et al., 2002), and the work done by Trost et al. (2012). Age may also be influencing participant’s emotional perception of each composition. Music is perceived more positively with increasing age, regardless of mode, key, and tempo (Cohrdes et al., 2020). This may result in older adults finding a wider array of music, including those composed in the minor mode (like our sad composition), to be happier than what is perceived by younger adults.

The sad music could also have been rated more neutrally by our older adult participants because of differences in exposure to the composition style of each musical composition. For instance, our non-musician participants likely have more experience with the happy music’s composition style as its style, 19^th^ Century Romanticism, is one we are accustomed to listening to in movies and commercials in the United States. Similarly, participants’ decreased experience with the sad music composition’s style may have caused them to perceive the sad music as more surprising, resulting in a more neutral emotional rating. These two explanations could explain why we did not observe more robust changes in attention between the happy and sad music listening groups. Future research, with a more balanced participant sample, is needed to better understand how middle and older aged adults perceive emotion in music, and how their perceptions may be modulated by gender and different musical features. A more balanced participant sample will also allow future studies to address whether the observed changes in attention following music listening differ when stratified by variables such as education and gender (see Supplementary material for exploratory analyses looking at the influence of education, age, and gender on the relationships between happy music and alerting and sad music and executive control in the current participant sample). It would also be useful for research to explore whether music listening similarly impacts attention in clinical populations which show impairments in alerting, orienting, and executive control (e.g., stroke).

While we find that happy and sad music both improve different aspects of attention, additional research is needed to investigate what role tempo and composition mode play in improving attention in older adults as we did not vary these features beyond the specified pairings (major-fast, minor-slow). We therefore cannot distinguish whether composition mode (major vs. minor), tempo (fast vs. slow), or the combination was inducing the observed changes in alerting and executive control. Similarly, other aspects of music not controlled for in this study including timbre, tonality, harmony, and composition key (to name a few) could also be contributing to the observed changes in attention. While a complete discussion of these aspects of music and their relationship to attention is beyond the scope of this study, it is important to note that our results may not generalize to all music written in the major mode with a fast tempo or minor mode with a slow tempo as it is possible that other musical properties are driving changes in attention. However, we speculate that mode and tempo may be important contributors to the observed changes in attention as the major mode and fast tempo and minor mode and slow tempo pairings are readily associated with feelings of happiness and sadness, and high and low arousal in non-musicians (Husain et al., 2002; Webster and Weir, 2005; Gabrielsson and Lindström, 2010; Hunter et al., 2010; Trost et al., 2012). Nonetheless, future work is needed to systematically study how aspects of music, beyond mode and tempo, modulate older adults’ attention.

### 4.4. Conclusion

Our results suggest that listening to both happy and sad music impacts middle-aged and older adults’ attention. More specifically, we found that happy music increases alerting attention, particularly on trials which contain conflict. Orienting attention also appeared to be modulated by happy music within incongruent trials only. These findings align with the arousal-mood hypothesis and provide further evidence that happy music, written in the major mode with a fast tempo, increases cognitive performance by increasing arousal (or alertness). Unexpectedly, sad music, written in the minor mode with a slow tempo, appears to improve executive control performance, possibly by priming the participant to focus their attention internally, thus aiding conflict resolution. While future studies are needed to replicate our findings and further explore the relationship between attention and other aspects of music beyond mode and tempo, our findings indicate that music written in the major mode with a fast tempo (happy) and minor mode with a slow tempo (sad) acutely modulate different aspects of attention.

## Data availability statement

The datasets presented in this study can be found in online repositories. The names of the repository/repositories and accession number(s) can be found at: https://osf.io/gf3km/.

## Ethics statement

The studies involving human participants were reviewed and approved by Midwestern University’s Institutional Review Board. The patients/participants provided their written informed consent to participate in this study.

## Author contributions

ND and AL was involved in experimental design, participant recruitment and testing, data analysis, and manuscript writing. SB, NJ, and IR were involved in experimental design, data analysis, and manuscript editing. All authors contributed to the article and approved the submitted version.

## Funding

This research was supported by Midwestern University.

## Conflict of interest

The authors declare that the research was conducted in the absence of any commercial or financial relationships that could be construed as a potential conflict of interest.

## Publisher’s note

All claims expressed in this article are solely those of the authors and do not necessarily represent those of their affiliated organizations, or those of the publisher, the editors and the reviewers. Any product that may be evaluated in this article, or claim that may be made by its manufacturer, is not guaranteed or endorsed by the publisher.

## Supplementary material

The Supplementary material for this article can be found online at: https://www.frontiersin.org/articles/10.3389/fpsyg.2023.1029773/full#supplementary-material

## Footnotes

^1^The invalid cues were not included in the analysis as they are not used in the calculation of any of the attentional subsystems. However, the means and standard deviations are included for completeness (Table 2).
