# Supplementary material for: Happy and sad music acutely modulate different types of attention in older adults
Source: Front Psychol. 2023 Jan 26;14:1029773. doi: 10.3389/fpsyg.2023.1029773 (PMC9909555; doi:10.3389/fpsyg.2023.1029773)
Supplement: Supplementary file 1 [file Data_Sheet_1.docx]

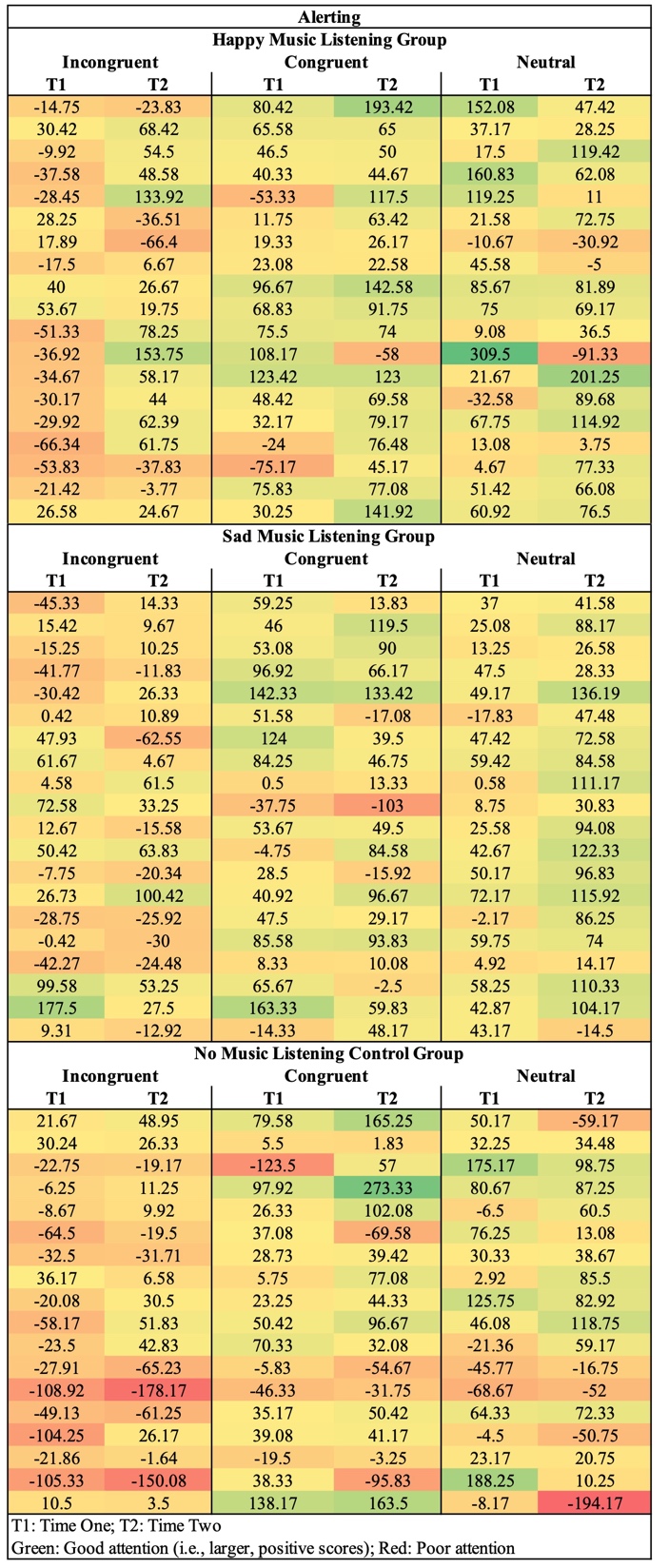


**Supplementary Figure 1.** Individual variation in how alerting (reaction time difference score for no cue – double cue) changes within incongruent, congruent, and neutral trials from time one to time two for each participant. Green shading represents better attention and red shading poorer attention. The individual data depicts a similar pattern to the whole-group analysis: alerting attention is relatively poor for all participants within incongruent trials at time one and improves at time two for the happy music listening group.


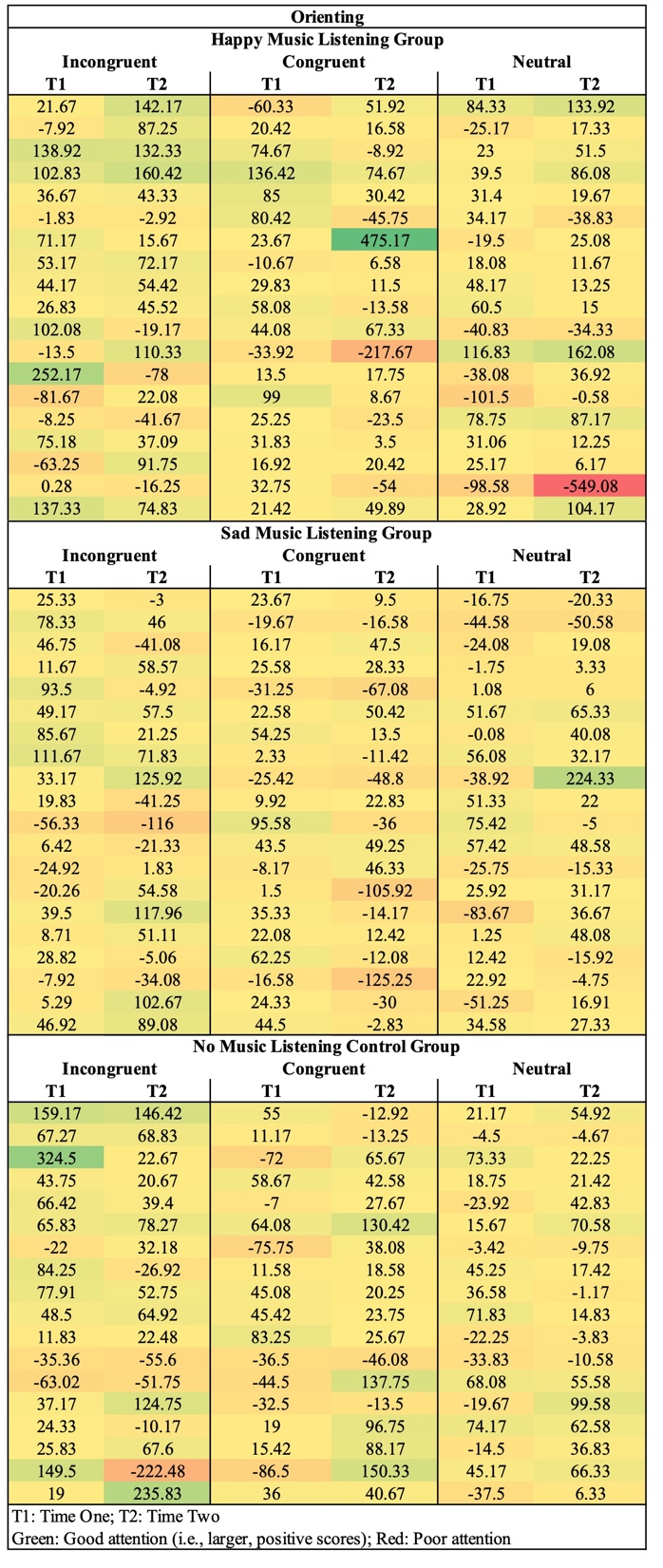


**Supplementary Figure 2.** Individual variation in how orienting (reaction time difference score for center cue – valid spatial cue) changes within incongruent, congruent, and neutral trials from time one to time two for each participant. Green shading represents better attention and red shading poorer attention. The individual data suggests that happy music may help to maintain orienting attention on the most challenging, incongruent trials.

**Exploratory analyses looking at the influence of education, age, and gender on the relationships between happy music and alerting and sad music and executive control.**

High education was defined as having at least a bachelor’s degree and low education as having less than a bachelor’s degree. Middle-age was defined as 59 years or younger and older-age as 60 years or older. The following results should be interpreted with caution given the small sample size and overall homogeneity of the participant sample.

**Happy Music and Alerting.** Seven participants had low education and 12 had high education; 8 participants were middle-aged and 11 participants older-aged; 3 participants were male and 16 were female. Attention across time did not interact with education (F(1,17)=0.002, *p=*.96), age (F(1,17)=1.62, *p=*.22), or gender (F(1,17)=2.67, *p=*.12).

**Sad Music and Executive Control.** Six participants had low education and 14 had high education; 11 participants were middle-aged and 9 participants older-aged; 3 participants were male and 17 were female. Attention across time did not interact with education (F(1,18)=0.004, *p=*.95), age (F(1,18)=1.20, *p=*.29), or gender (F(1,18)=0.002, *p=*.97).
